# Genetic polymorphism in melatonin receptor 1A and arylalkylamine N-acetyltransferase and its impact on seasonal reproduction in Egyptian sheep breeds

**DOI:** 10.5194/aab-61-505-2018

**Published:** 2018-12-20

**Authors:** Hager A. Fathy, Eman M. Gouda, Jehan A. Gafer, Mona K. Galal, Amira M. Nowier

**Affiliations:** 1 Biotechnology unit, Animal Reproduction Research Institute, Giza, Egypt; 2 Department of Biochemistry and Chemistry of Nutrition, Faculty of Veterinary Medicine, Cairo University, Giza, Egypt; 3 Biotechnology Research Department, Animal Production Research Institute, Dokki, Egypt

## Abstract

This study was carried out to detect polymorphisms in the melatonin receptor 1A (MTNR1A) and arylalkylamine N-acetyltransferase (AA-NAT) genes and their association with reproductive traits. Blood samples of 126 animals from three Egyptian sheep breeds were collected. DNA was extracted and subjected to PCR restriction fragment length polymorphism (RFLP) analysis using the RsaI and SmaI enzymes. Two alleles (C and T) and three genotypes (CC, CT and TT) for MTNR1A and for AA-NAT (A and G; GG, GA and AA) were detected. The alleles C and A and the genotypes CT and GA showed the highest frequencies for the MTNR1A and AA-NAT genes, respectively. Association analysis of the MTNR1A single nucleotide polymorphism (SNP) with ewe reproductive traits revealed significant associations in the Ossimi and Rahmani breeds with age at first lambing, and the C allele seemed to be the favorable allele. The results for the AA-NAT SNP demonstrated significant correlations in Ossimi with age at first lambing and litter size and in Rahmani with lambing interval; the G allele seemed to be the desirable allele. In the first conception season, ewes carrying CT exhibited a significantly lower age of first lambing in the unfavorable season. Additionally, GG ewes exhibited a significantly lower age of first lambing in the early favorable season, followed by the unfavorable season. To the best of our knowledge, this is the first study of these associations in Egyptian sheep breeds. In conclusion, the polymorphisms revealed in this study could be used as genetic markers to improve reproductive efficiency during the unfavorable season, and the obtained desirable genotypes could be considered in new genetic selection schemes.

## 1 Introduction

Sheep are considered important farm animals and contribute significantly to the livelihood of human populations. Increasing the human population creates more demand for these animals and their products. This demand can efficiently be met by increasing the reproductive capacity and productivity of these animals (Kosgey and Okeyo, 2007). Rahmani, Ossimi and Barki are the main sheep breeds in Egypt and contribute approximately 6 % of total red meat production (Galal et al., 2005). Sheep reproduction is widely known to involve marked seasonality (Rosa and Bryant, 2003), and this seasonal variation in fertility is a major obstacle to increasing the intensity of sheep production. Satisfactory reproductive performance is mainly limited by the need to lengthen the breeding season to encompass unfavorable breeding seasons (Bartlewski et al., 2011). Teyssier et al. (2011) stated that changes in day length may act as a major factor controlling seasonal changes in estrous activity in sheep breeds with maximal reproductive activity during short days. The identification of quantitative trait loci (QTL) and the implementation of marker-assisted selection (MAS) could assist in selection schemes to shorten the anestrous season and open a new chapter in forecasting and controlling the fertility of sheep (Petrovic et al., 2012). Melatonin is called the “hormone of darkness” because its production is controlled by day and night alteration (Rosa and Bryant, 2003). Short photoperiods positively influence melatonin levels, and these increasing melatonin levels stimulate the pituitary gland to release follicle-stimulating hormone and luteinizing hormone (Falk, 2013). Melatonin exerts its influence via two specific receptors named 1A and 1B. However, melatonin receptor 1A (MTNR1A) is the main receptor that mediates the reproductive and circadian actions of melatonin in sheep. Polymorphisms in the MTNR1A gene have been documented to be associated with seasonal reproduction in various breeds of sheep (Carcangiu et al., 2009a; Ahmad et al., 2015), goat (Ağaoğlu et al., 2015), pig (Ramírez et al., 2009), deer (Yang et al., 2014) and buffalo (Barbosa et al., 2017). Arylalkylamine N-acetyltransferase (AA-NAT) is called “the Timezyme” because it plays a unique role in vertebrate time-keeping (Klein, 2007) and is considered the rate-limiting enzyme in melatonin biosynthesis. Therefore, any polymorphism in the AA-NAT gene may contribute to the variability in melatonin production and influence seasonal estrus response in the sheep population (Koike et al., 2013; Öner et al., 2014). The AA-NAT gene has been associated with seasonal reproductive patterns in the Jining Grey goat of China (Chu et al., 2013), Indian goat breeds (Sharma et al., 2015) and Chinese sheep breeds (Ding-ping et al., 2012). In Egypt, no adequate studies have been carried out to determine the reproductive seasonality of Egyptian native sheep breeds as it relates to polymorphisms in these candidate genes and their expected effects on ewe reproductive performance. The objectives of the present study were, first, to identify polymorphisms of MTNR1A and AA-NAT genes in three Egyptian sheep breeds; and second, to investigate the associations between the MTNR1A gene and AA-NAT genes with ewe reproductive performance traits. This achievement could provide a theoretical basis for genetically controlling ovine estrus and lay a foundation for both changing ovine estrous seasonality and improving reproductive performance by means of gene selection.

## 2 Material and methods

### 2.1 Animals

The present study was conducted on a total of 126 animals belonging to three Egyptian sheep breeds: Ossimi, n=66; Rahmani, n=41 and Barki, n=19 (raised in the Animal Production Research Station). Age at first lambing (AFL), lambing interval (LI), litter size (LS) and fertility rate were used to evaluate reproductive performance. The seasons of conception were defined as early favorable (EF; September to November), late favorable (LF; December to February), or unfavorable (UF; March to August). The animal experiment was conducted after approval of the Institutional Animal Care and Use Committee, Cairo University (CU-IACUC), with approval number CUIIS5117.

### 2.2 Blood sampling and DNA extraction

Blood samples were collected by jugular vein puncture into EDTA vacuum tubes and kept at -20 ${}^{\circ }$C until use. Genomic DNA was extracted using a genomic DNA extraction purification kit (Quick-gDNA MiniPrep^™^, Zymo Research, USA) according to the manufacturer's instructions. DNA quantity and purity for each sample were assessed by spectrophotometer.

### 2.3 Polymerase chain reaction for MTNR1A and AA-NAT

A PCR fragment of exon 2 of the ovine MTNR1A gene sequence (GenBankU14109) was amplified with specific primers as described by Messer et al. (1997) with the following sequences: forward 5${}^{\prime }$-TGTGTTTGTGGTGAGCCTGG-3${}^{\prime }$ and reverse 5${}^{\prime }$-ATGGAG AGGGTTTGCGTTTA-3${}^{\prime }$. The AA-NAT gene (GenBankJX444551.1) amplicon includes part of exon 1, intron 1, whole exon 2, intron 2 and part of exon 3. The sequence was amplified with specific primers as described by Ding-ping et al. (2012) with the following sequences: forward 5${}^{\prime }$-AGCGTCCACTGCCTGAAAC-3${}^{\prime }$ and reverse 5${}^{\prime }$-GGGATGGAAGCCAAACCTC-3${}^{\prime }$ (Invitrogen by Thermo Fisher Scientific, EU). The amplifications were performed with a thermocycler (nexus gradient Eppendorf^™^^®^ AG 2231 Hamburg, Germany) in a total volume of 25 µL containing 1 µL of DNA, 1× master mix (Thermo Fisher Scientific, EU), 10 pmol µL${}^{-1}$ of each primer and nuclease-free water (NFW) with the addition of 1 µL bovine serum albumin (2.5 mg mL${}^{-1}$) with the following temperature profiles. For the MTNR1A gene, an initial denaturation at 94 ${}^{\circ }$C for 5 min; followed by 35 cycles of denaturation at 95 ${}^{\circ }$C for 1 min, annealing at 62 ${}^{\circ }$C for 1 min and elongation at 72 ${}^{\circ }$C for 1 min; and a final elongation at 72 ${}^{\circ }$C for 10 min. For the AA-NAT gene, an initial denaturation at 94 ${}^{\circ }$C for 5 min; followed by 35 cycles of denaturation at 95 ${}^{\circ }$C for 45 s, annealing at 60 ${}^{\circ }$C for 45 s and elongation at 72 ${}^{\circ }$C for 2 min; and final elongation at 72 ${}^{\circ }$C for 10 min. The PCR products were detected on 1.5 % ethidium-bromide-stained agarose gels by electrophoresis.

**Figure 1:**
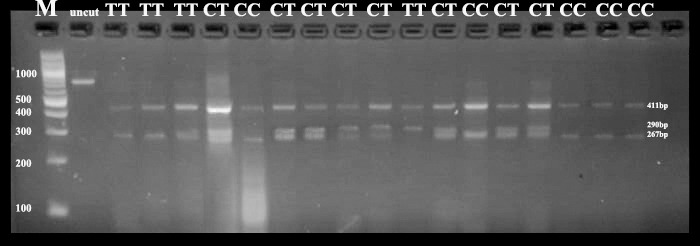
Agarose gel electrophoresis (3 %) showing PCR RFLP of exon 2 of MTNR1A using the RsaI enzyme. M: 100 bp DNA ladder; uncut, 824 bp. Lanes represent the genotypes CC (411, 267 bp), TT (411, 290 bp) and CT (411, 290, 267 bp).

### 2.4 Restriction fragment length polymorphism (RFLP) analysis

The PCR product of the MTNR1A gene was digested by the RsaI enzyme (Thermo Scientific Fast Digest, Lithuania, EU) according to Saxena et al. (2015a). The reaction was conducted in a 30 µL volume containing 10 µL of amplicon, 1 µL of enzyme, 2 µL of 10× buffer and 17 µL NFW at 37 ${}^{\circ }$C for 90 min followed by deactivation at 65 ${}^{\circ }$C for 20 min. The PCR product of the AA-NAT gene was digested by the SmaI enzyme (New England Biolabs CutSmart^™^, MA, USA) according to Ding-ping et al. (2012). The reaction was conducted in a 40 µL volume containing 16 µL of amplicon, 1 µL of enzyme, 5 µL of 10× buffer and 18 µL of NFW at 25 ${}^{\circ }$C for 90 min followed by deactivation at 65 ${}^{\circ }$C for 20 min. The digestion results were visualized by 3 % agarose gel electrophoresis and staining with ethidium bromide.

### 2.5 Statistical analysis

Estimates of genotypic and allelic frequencies, unbiased expected heterozygosity analyses and Hardy–Weinberg equilibrium tests for each breed population were carried out using GENEPOP software, version 4.2, according to Yeh et al. (1999). To test the associations of different conformational patterns with reproductive traits, including AFL, LI, LS and fertility, the preliminary data analysis was subjected to a two-way analysis of variance, with conformation patterns, first conception season, season of lambing, and parity as fixed effects using the general linear model (GLM) procedure of the Statistical Analysis System (SAS, 2002) program, version 9.1. The following linear model for the reproductive traits studied was used:

$$\begin{array}{cc} \mathrm{Yijklmn} & =\mu \;+\;\mathrm{Bi}\;+\;\mathrm{Gj}\;+\;\mathrm{Fk}\;+\;\mathrm{Sl}\;+\;\mathrm{Pm}\;+\;(\mathrm{GF})jk\\ & +\;(\mathrm{GS})jl\;+\;\mathrm{eijklmn}, \end{array}$$

where Yijklmn: measurements of reproductive traits, μ: overall mean, Bi: fixed effect of the ith breed (1 = Ossimi, 2 = Rahmani, 3 = Barki), Gj: fixed effect of the jth conformation pattern (1, 2, 3), Fk: fixed effect of the kth first conception season (1 = LF, 2 = UF, 3 = EF), Sl: fixed effect of the season of lambing (1 = LF, 2 = UF, 3 = EF), Pm: fixed effect of the ith parity (1, 2 … 13), (GF) jk: fixed effect of the jth conformation pattern nested within the kth first conception season. (GS)jl: fixed effect of the jth conformation pattern nested within the lth season of lambing, eijklmn: random residual errors assumed to be normally distributed with mean = 0 and variance =σ2e.

## 3 Results

### 3.1 PCR amplification of MTNR1A and AA-NAT gene fragments

A DNA fragment with an expected size of 824 bp, corresponding to exon 2 of the MTNR1A gene, was obtained from sheep DNA using specific primers. Additionally, the AA-NAT gene was successfully amplified, giving rise to an 1142 bp amplicon.

### 3.2 RFLP and genotyping of MTNR1A exon 2 using the RsaI enzyme

The results of the RsaI enzyme digestion revealed three genotypes: CC (411 bp, 267 bp), CT (411 bp, 290 bp, 267 bp) and TT (411 bp, 290 bp) (Fig. 1). All three genotypes were identified in the Ossimi and Rahmani breeds, while only two genotypes (CC and CT) were detected in the Barki breed.

**Table 1:** Genotypes and allele frequencies of the MTNR1A SNP in Egyptian sheep breeds. Where N is the total number of animals in each breed; UHe is the unbiased expected heterozygosity; number in parentheses is the no. of genotype observed; ns represents non-significant.

Breeds	Observed genotype frequencies			Expected genotype frequencies			Allele frequencies		UHe	Hardy–Weinberg equilibrium	
	CC	CT	TT	CC	CT	TT	C	T		$\chi^{2}$ test	P value
Ossimi N=66	0.409 (27)	0.455 (30)	0.136 (9)	0.405	0.463	0.132	0.636	0.364	0.466	0.021	0.885${}^{\mathrm{ns}}$
Rahmani N=41	0.260 (11)	0.439 (18)	0.293 (12)	0.238	0.500	0.262	0.488	0.512	0.506	0.605	0.437${}^{\mathrm{ns}}$
Barki N=19	0.632 (12)	0.368 (7)	0 (0)	0.666	0.301	0.034	0.816	0.184	0.309	0.969	0.325${}^{\mathrm{ns}}$
Total N=126	0.397 (50)	0.437 (55)	0.167 (21)	0.378	0.474	0.148	0.615	0.385	0.476	0.770	0.380${}^{\mathrm{ns}}$

### 3.3 Genotypes and allele frequencies for the MTNR1A receptor gene

The allelic and genotypic frequencies of the MTNR1A SNP are shown in Table 1. Overall, the frequencies of allele C (0.615) and genotype CT (0.437) were obviously the highest. The estimated mean observed and expected heterozygosity values at the MTNR1A–RsaI marker site in all sheep breeds were equivalent (0.437 for observed genotype frequencies and 0.474 for expected genotype frequencies). The genetic diversity estimated by unbiased heterozygosity (UHe) was highest in Rahmani (0.506), followed by Ossimi (0.466) and Barki (0.309). The exact P values obtained from the $\chi^{2}$ test in all populations were consistent with Hardy–Weinberg equilibrium in all investigated breeds.

### 3.4 RFLP and genotyping of the AA-NAT exon 3 polymorphism using the SmaI enzyme

**Figure 2:**
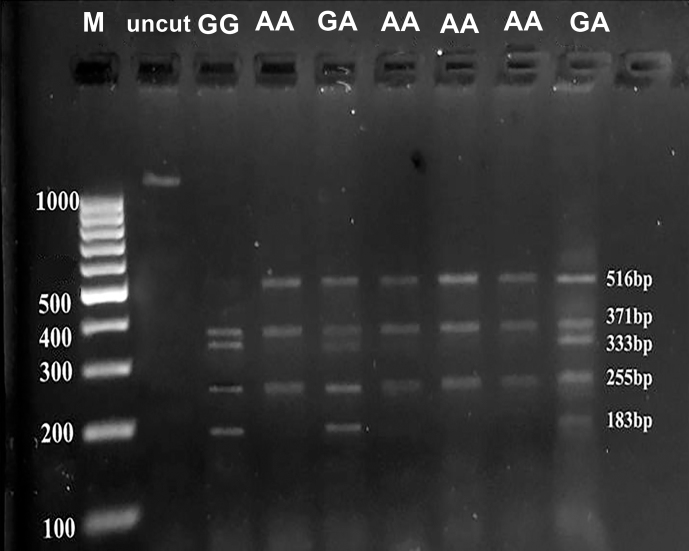
Agarose gel electrophoresis (3 %) showing PCR RFLP and genotyping of the AA-NAT exon 3. M: 100 bp DNA ladder; uncut, 1142 bp. Lanes represent the genotypes GA (516, 371, 333, 255, 183 bp), GG (371, 333, 255, 183 bp) and AA (516, 371, 255 bp).

The results of the SmaI enzyme assay revealed three genotypes: AA (516 bp, 371 bp, 255 bp), GG (371 bp, 333 bp, 255 bp, 183 bp) and GA (516 bp, 371 bp, 333 bp, 255 bp, 183 bp) (Fig. 2). All three genotypes were identified in the Ossimi and Rahmani breeds, while no GA genotype was detected in the Barki breed (Fig. 2).

**Table 2:** Genotypes and allele frequencies of the AA-NAT SNP in Egyptian sheep breeds. Where N is the total number of animals in each breed; UHe is the unbiased expected heterozygosity; number in parentheses is the no. of genotype observed; ns represents non-significant.

Breeds	Observed genotype frequencies			Expected genotype frequencies			Allele frequencies		UHe	Hardy–Weinberg equilibrium	
	GG	GA	AA	GG	GA	AA	G	A		$\chi^{2}$ test	P value
Ossimi N=66	0.090 (6)	0.333 (22)	0.576 (38)	0.066	0.382	0.551	0.258	0.742	0.385	1.089	0.297${}^{\mathrm{ns}}$
Rahmani N=41	0.244 (10)	0.585 (24)	0.171 (7)	0.288	0.497	0.215	0.537	0.463	0.503	1.285	0.257${}^{\mathrm{ns}}$
Barki N=19	0 (0)	0.842 (16)	0.158 (3)	0.177	0.488	0.335	0.421	0.579	0.501	10.050	0.002${}^{*}$
Total N=126	0.127 (16)	0.492 (62)	0.381 (48)	0.139	0.468	0.393	0.373	0.627	0.470	0.340	0.560${}^{\mathrm{ns}}$

${}^{*}$ at the p 0.05 level.

### 3.5 Genotyping and allelic frequency for the AA-NAT polymorphism

The allelic and genotypic frequencies of the AA-NAT SNP are shown in Table 2. Overall, allele A (0.627) and genotype GA had the highest frequency (0.492). The estimated mean observed and expected heterozygosity values for the AA-NAT–SmaI marker site in all sheep breeds were equivalent (0.492 for observed genotype frequencies and 0.468 for expected genotype frequencies). The results of the genetic diversity estimate by UHe showed the highest degree in Rahmani (0.503), followed by Barki (0.501) and Ossimi (0.385). The exact P values obtained from the $\chi^{2}$ test in Ossimi and Rahmani confirm consistency with Hardy–Weinberg equilibrium. However, the Barki breed was found to deviate from it (P=0.002).

**Table 3:** Effect of the MTNR1A SNP on reproductive traits in Egyptian sheep breeds. Where is the number of observed genotypes; LSM represents least-square mean; SE represents standard error; N is the number of observed genotypes.

Breeds	Genotype	Age at first lambing				Lambing interval			Litter size			Fertility		
		N	LSM	±	SE	LSM	±	SE	LSM	±	SE	LSM	±	SE
Ossimi	CC	27	1006.60	±	33.25	453.30	±	39.09	1.01	±	0.03	0.58	±	0.08
	CT	30	953.72	±	36.83	493.60	±	5.47	1.07	±	0.04	0.59	±	0.08
	TT	9	1176.55	±	56.59	447.20	±	78.75	1.00	±	0.07	0.57	±	0.13
	P value	66	0.001${}^{**}$			0.66			0.24			0.97		
Rahmani	CC	11	806.73	±	58.26	407.09	±	40.01	1.12	±	0.09	0.44	±	0.15
	CT	18	855.01	±	40.32	377.36	±	26.39	0.98	±	0.06	0.52	±	0.10
	TT	12	960.95	±	56.85	300.28	±	37.71	0.99	±	0.08	0.65	±	0.15
	P value	41	0.04${}^{*}$			0.06			0.24			0.39		
Barki	CC	12	579.99	±	18.30	347.57	±	51.25	1.00	±	0.00	1.00	±	0.00
	CT	7	617.47	±	18.24	364.68	±	45.79	1.00	±	0.00	1.00	±	0.00
	TT	0	–	±		–	±	–	–	±	–	–	±	–
	P value	19	0.06			0.74			–			–		
All breeds	P value	126	0.04${}^{*}$			0.59			0.10			0.93		

${}^{*}$ is significant at the p 0.05 level, and ${}^{**}$ is significant at the p 0.01 level.

### 3.6 Impact of the MTNR1A polymorphism on sheep reproductive traits

The results of the association analysis of the MTNR1A SNP with reproductive traits revealed a significant association (P=0.04 and P>0.001) between genotypes and age at first lambing in Rahmani and Ossimi, respectively, where CC Rahmani and CT Ossimi ewes were characterized by the lowest age at first lambing. However, in the Barki breed, there were no significant differences between the two detected genotypes and any studied traits. Across the whole population, there were significant differences (P=0.04) associated with the detected genotypes for AFL, while there were no significant differences associated with other traits (Table 3).

**Table 4:** Effect of the MTNR1A SNP on reproductive traits in Egyptian sheep breeds in their first conception season. Where FCS is the first conception season; N is the number of observed genotype; LSM is the least-square mean; SE represents standard error; LF represents late favorable season; UF represents unfavorable season; EF represents early favorable season.

Genotype	FCS	Age at first lambing				Lambing interval				Litter size				Fertility			
		N	LSM	±	SE	N	LSM	±	SE	N	LSM	±	SE	N	LSM	±	SE
CC	LF	39	832.09	±	45.11	20	419.45	±	49.51	27	0.96	±	0.05	39	0.68	±	0.10
	UF	54	709.72	±	42.31	21	523.37	±	50.76	33	1.03	±	0.04	54	0.67	±	0.10
	EF	42	698.87	±	44.31	21	371.16	±	48.18	25	0.95	±	0.05	42	0.66	±	0.10
CT	LF	24	808.65	±	49.29	11	370.96	±	57.32	16	0.96	±	0.05	24	0.74	±	0.11
	UF	64	692.97	±	41.61	20	505.05	±	50.33	35	1.02	±	0.04	64	0.68	±	0.10
	EF	65	742.70	±	39.63	37	430.48	±	50.33	40	0.96	±	0.04	65	0.66	±	0.10
TT	LF	9	716.30	±	82.49	3	331.49	±	111.02	3	1.42	±	0.11	9	0.62	±	0.19
	UF	30	915.82	±	51.46	11	396.68	±	60.51	17	0.94	±	0.05	30	0.71	±	0.12
	EF	11	924.00	±	73.21	5	411.57	±	81.57	7	0.93	±	0.07	11	0.69	±	0.17
	P value	0.01${}^{*}$				0.41				0.002${}^{*}$				0.98			

${}^{*}$ is significant at the p 0.05 level.

The effect of the MTNR1A SNP on reproductive traits with reference to the first conception season in Egyptian sheep is presented in Table 4. Significant associations were found with AFL (P>0.01) and LS (P=0.002), and the table demonstrates that ewes carrying the CT genotype and conceiving for the first time in the unfavorable season had the lowest mean AFL, followed by those with the CC genotype conceiving in the early favorable season. With reference to LS, TT individuals seemed to tend towards higher LS in the late favorable conception season, followed by CC individuals in the unfavorable season. No significant difference was found for lambing interval or fertility rate.

**Table 5:** Effect of the AA-NAT SNP on reproductive traits in Egyptian sheep breeds. Where N is the number of observed genotypes; LSM represents least-square mean; SE represents standard error.

Breeds	Genotype	N	Age at first lambing			Lambing interval			Litter size			Fertility		
			LSM	±	SE	LSM	±	SE	LSM	±	SE	LSM	±	SE
Ossimi	GG	6	933.68	±	62.02	550.94	±	72.80	1.20	±	0.05	0.77	±	0.13
	GA	22	1072.31	±	41.31	580.43	±	48.44	1.05	±	0.04	0.48	±	0.09
	AA	38	987.04	±	31.63	432.29	±	32.43	1.01	±	0.03	0.61	±	0.07
	P value	66	0.02${}^{*}$			0.01${}^{**}$			0.01${}^{**}$			0.08		
Rahmani	GG	10	783.98	±	56.80	359.34	±	38.51	0.94	±	0.08	0.55	±	0.14
	GA	24	899.06	±	41.00	354.80	±	24.24	1.03	±	0.06	0.57	±	0.10
	AA	7	882.34	±	69.71	522.39	±	57.53	0.97	±	0.13	0.21	±	0.17
	P value	41	0.14			0.02${}^{*}$			0.54			0.09		
	GG	0	–	±	–	–	±	–	–	±	–	–	±	–
Barki	GA	16	601.66	±	15.85	353.97	±	40.64	1.00	±	0.00	1.00	±	0.00
	AA	3	559.03	±	32.04	407.23	±	78.75	1.00	±	0.00	1.00	±	0.00
	P value	19	0.16			0.47			–			–		
All breeds	P value	126	0.03${}^{*}$			0.45			0.35			0.22		

${}^{*}$ is significant at the p 0.05 level, and ${}^{**}$ is significant at the p 0.01 level.

### 3.7 Impact of the AA-NAT polymorphism on sheep reproductive traits

The association analysis of the AA-NAT gene polymorphism with ewe reproductive traits is shown in Table 5. The results showed that the Ossimi breed had a significant association between genotype and AFL (P=0.02), LS (P<0.01) and LI (P<0.01), where the lowest age at first conception and the highest LS were recorded in GG individuals.

On the other hand, AA Ossimi individuals showed the lowest lambing intervals, and the result was significant (P>0.01). In the Rahmani breed, GA individuals showed the shortest lambing interval (P=0.02). In the Barki breed, there were no significant differences between different genotypes and traits. Overall, the population showed a significant association (P=0.03) for AFL only.

**Table 6:** Effect of the AA-NAT SNP on reproductive traits in Egyptian sheep breeds in their first conception season. Where N is the number of observed genotypes; LSM represents least-square mean; SE represents standard error; LF represents late favorable season; UF represents unfavorable season; EF represents early favorable season.

Genotype	FCS	Age at first lambing				Lambing interval				Litter size				Fertility			
		N	LSM	±	SE	N	LSM	±	SE	N	LSM	±	SE	N	LSM	±	SE
GG	LF	2	676.16	±	155.24	1	247.31	±	175.81	2	0.96	±	0.12	2	1.18	±	0.36
	UF	22	662.37	±	57.94	8	377.90	±	79.20	13	1.14	±	0.06	22	0.81	±	0.13
	EF	22	578.54	±	56.84	10	378.05	±	64.25	12	0.96	±	0.06	22	0.68	±	0.13
GA	LF	45	759.96	±	39.12	24	403.43	±	42.21	31	1.04	±	0.04	45	0.71	±	0.09
	UF	71	806.94	±	39.15	25	453.08	±	42.94	44	1.06	±	0.04	71	0.68	±	0.09
	EF	52	796.07	±	42.55	29	410.00	±	44.34	30	1.00	±	0.04	52	0.59	±	0.10
AA	LF	25	873.19	±	52.93	9	358.71	±	65.34	13	0.98	±	0.06	25	0.64	±	0.12
	UF	55	669.77	±	44.04	19	499.15	±	51.31	28	1.00	±	0.05	55	0.62	±	0.10
	EF	44	722.56	±	45.43	24	344.06	±	48.35	30	0.98	±	0.04	44	0.73	±	0.10
	P value	0.003${}^{*}$				0.37				0.39				0.33			

${}^{*}$ at the p 0.05 level.

The relationships between both isoforms of the AA-NAT gene and reproductive traits with reference to conception season are presented in Table 6. A significant difference associated with age at first lambing was detected (P=0.003), where GG ewes conceiving for the first time in the early favorable season or even in the unfavorable season had fewer days to reach AFL. No significant associations were found with the remaining reproductive traits.

## 4 Discussion

Seasonal variation in fertility is an important factor limiting the efficiency of sheep production, resulting in the occurrence of births and milk production in specific periods of the year (Trecherel et al., 2010). The identification of polymorphisms in genes affecting nonseasonal reproduction, such as the MTNR1A and AA-NAT genes, could open a new chapter in predicting and controlling the sexual activity of sheep (Petrovic et al., 2012). MTNR1A is thought to be the main receptor involved in the regulation of seasonal reproductive activities in mammals (Dubocovich et al., 2003). Exon 2 of the MTNR1A gene is known to be highly polymorphic, and one of these polymorphisms involves a restriction site for the RsaI enzyme, 606 C > T (Saxena et al., 2015a). The AA-NAT gene is considered an ideal gene to investigate possible SNPs that could clarify the large individual variations in serum melatonin levels and may aid in controlling seasonal reproductive activity (Klein, 2007; Barclay et al., 2010). The results of PCR RFLP revealed three genotypes (CC, CT and TT) for exon 2 of the MTNR1A gene and three (GG, GA and AA) for exon 3 of the AA-NAT gene, and two alleles were detected for each gene in all analyzed breeds, which emphasizes that selection has not eliminated any of the alternative forms of the MTNR1A gene (Notter and Cockett, 2005).

The allelic frequency of the MTNR1A gene obtained in this study revealed that allele C was predominant in both the Ossimi and Barki breeds. This result was in accordance with the previous study of Mura et al. (2014) on Sarda sheep, Hristova et al. (2012) on the Bulgarian Local Karnobatska (LKNB) breed, Moradi et al. (2014) on Zel and Naeini lamb, and Giantsis et al. (2016) on a local Greek sheep breed. However, quite a different result was obtained in the Rahmani breed, where allele T was predominant. Similar results were recorded by Notter and Cockett (2005) in Suffolk and Israel Awassi sheep, Mateescu et al. (2009) in Dorest sheep and Saxena et al. (2015a) in subtemperate sheep. Concerning the genotypic distribution of the MTNR1A gene, the CT genotype had the highest frequencies in Ossimi and Rahmani, which were found to be similar to the results of Falk (2013) on Swedish (Roslag and fine wool) sheep breeds. Similarly, Hristova et al. (2012) reported the same result in Bulgarian sheep breeds. However, in the Barki breed, the CC genotype was more frequent. The same result was reported by Giantsis et al. (2016) in a local Greek sheep breed, Saxena et al. (2015b) in the tropical sheep breeds of India, and Avanus and Altinel (2016) in Kıvırcık breed ewes. The TT homozygous genotype had the lowest frequencies in Ossimi and Rahmani and was not represented in the Barki breed, probably due to the low sample size. A similar observation was previously reported by Hristova et al. (2012) who found that the TT genotype was not represented in the Bulgarian LKNB sheep breed. In contrast, the TT genotype was predominant in Dorset ewes (Mateescu et al., 2009). The exact P values obtained from the $\chi^{2}$ test in all populations confirm Hardy–Weinberg equilibrium in all investigated breeds. Therefore, we can postulate that these alleles' frequencies did not change over time. This result might be attributed to the absence of any effect of selection on this site (Moradi et al., 2014). Genetic diversity serves as a way for populations to adapt to changing environments. With more variation, some individuals in a population will possess alleles that are more suited for the environment. The population will continue for more generations because of the success of these individuals (Reed and Frankham, 2003). Rahmani had the highest level of genetic diversity as estimated by UHe, followed by Ossimi. Additionally, Hristova et al. (2012) reported a high degree of estimated average genetic diversity in the Breznishka, followed by Sofiiska (Elin-Pelinska), sheep breed. The estimated mean observed and expected heterozygosity values for the MTNR1A–RsaI marker site in all sheep breeds were equivalent. Therefore, the detected heterozygote deficit was low (inbreeding). This result is in accordance with the previous results of Hristova et al. (2012) in four Bulgarian sheep breeds.

The allelic distribution of the AA-NAT gene revealed that allele A was more frequent in Ossimi and Barki sheep, while allele G was more frequent in Rahmani sheep. These data were in accordance with the results obtained from Turkish sheep breeds (Öner et al., 2014). In contrast, a similar study carried out in Chinese sheep breeds revealed that the frequency of the G allele was higher in nonseasonal reproduction breeds while found quite lower in the seasonal reproduction breeds (Xinjiang Finewool sheep and Altay Fat-Rumped sheep; Ding-ping et al., 2012). Regarding the genotypic distribution of the AA-NAT gene, the GA and AA genotypes were detected in all breeds. However, the GG genotype was not observed in Barki sheep. Among the three studied breeds, the heterozygous GA genotype had the highest frequency in Rahmani and Barki. Similar results were observed in the Xinjiang Finewool sheep and Altay Fat-Rumped sheep breeds (Ding-ping et al., 2012). In the Ossimi breed, the AA homozygous genotype was more frequent. This finding was consistent with the results reported by Öner et al. (2014) in a Turkish sheep breed. However, the GG genotype was not observed in Barki sheep. Additionally, Ding-ping et al. (2012) reported that the AA genotype was not detected in Small Tailed Han and Dolang sheep. The exact P values obtained from the $\chi^{2}$ test in Ossimi and Rahmani confirmed accordance with Hardy–Weinberg equilibrium. However, the Barki breed deviated from Hardy–Weinberg equilibrium, possibly due to the low sample size. The estimated mean value of UHe as an estimator of genetic diversity was 0.463 in the examined Egyptian sheep population. Rahmani showed a higher level of genetic diversity, followed by Barki. In comparison to these breeds, Ossimi showed the lowest value for genetic diversity. Due to the rarity of the literature on the AA-NAT gene in sheep, it is difficult to compare our results in Egyptian sheep with those in other sheep breeds. According to the results reported in Table 3, there are significant correlations between different MTNR1A genotypes and AFL in Rahmani and Ossimi breeds, as CC Rahmani and CT Ossimi had the lowest least-square means; allele C seems to be the favorable allele. This effect may be due to decreased sensitivity to photoperiod (Chemineau et al., 2010). In the same context, Chu et al. (2006) showed a significantly higher allelic frequency of allele C in nonseasonal sheep breeds. Similarly, Carcangiu et al. (2009a) reported that the CC genotype was associated with year-round breeding performance in Sarda sheep breeds. Mura et al. (2014) demonstrated that ewes carrying the CC and CT genotypes had significantly higher fertility rates and fewer days between the introduction of rams and parturition. Additionally, Giantsis et al. (2016) reported that the CC genotype seemed to have an additive effect on sheep fertility with respect to out-of-season reproduction activity. Luridiana et al. (2016) found that the CC genotype had a better response to melatonin treatment in Sarda sheep. These results disagree with those of Martínez-Royo et al. (2012) who found that in the Rasa Aragonesa breed the T allele was associated with an early onset of reproductive activity in the spring. On the other hand, Barki sheep showed no significant association between the detected genotypes and AFL. The same observation was reported by Mateescu et al. (2009) in Dorest ewes. Additionally, Falk (2013) suggested a lack of relationship between MTNR1A polymorphism and reproductive seasonality in Swedish ewes. The mechanism by which the polymorphism in the MTNR1A gene affects out-of-season reproduction has not been established, because the polymorphisms evaluated in previous studies do not result in amino acid substitutions in the melatonin receptor; and thus, these different genotypes do not change the binding abilities of the receptor (Trecherel et al., 2010). One explanation could be that this polymorphism may be associated with other mutations in other sites of the nucleotide sequence or other genes closely linked with the MTNR1A gene, which could lead to changes in receptor functionality (Luridiana et al., 2016). Investigation of the impact of the AA-NAT SNP on reproductive traits indicated that GG Ossimi ewes tended to have lower AFL and higher LS. However, GA Rahmani sheep should be considered for selection due to their shorter lambing interval. In Barki ewes, no significant effect of genotypes on reproductive traits was obtained, and more investigation must be carried out. Ding-ping et al. (2012) reported that the genotype GG might be associated with nonseasonal reproduction, while the genotype GA might be associated with seasonal reproduction. This work also suggested that this novel mutation in exon 3 led to changes in amino acids (Arg > Gly), which likely had effects on AA-NAT protein structure, altering biological function, and might affect nonseasonal reproduction in sheep. In contrast, the AA genotype was predominant in Turkish sheep breeds (Öner et al., 2014). Notably, however, there is a pronounced drop in cycling activity during the spring and summer months in Egyptian sheep breeds (Aboul-Ela and Chemineau, 1990). The autumn and winter breeding seasons in local breeds had a significantly higher number of lambs born per ewe than the summer and spring breeding seasons (Ahmed, 2008). Therefore, it is important to plan lambing for unfavorable breeding seasons with the aim of continuous meat production throughout the year, especially in the months when lambing is less frequent. Thus, the possibility of correlation between both variants of the MTNR1A gene and reproductive traits in the first conception season was evaluated in this study. The results shown in Table 4 demonstrate that CT ewes that conceive for the first time in the unfavorable season have the lowest mean AFL. This result is in accordance with the report of Carcangiu et al. (2009b) who showed a strong link between heterozygous genotype and reproductive activity in different goat breeds. Thus, it can be hypothesized that ewes carrying one or more C alleles exhibit a shallow state of anestrus that increases their response to the ram effect (Carcangiu et al., 2011a). This supposition is in accordance with previous studies in other sheep breeds (Chu et al., 2006 and Carcangiu et al., 2009a). Thus, ewes that carry one or more C alleles seem to be more desirable or selectable for lowest AFL. The effect of the season of first conception on age at first lambing could be explained as follows: ewes that conceive in the unfavorable season are more likely to conceive sooner after lambing because their next breeding season will be in the early favorable season (Mateescu et al., 2009). With reference to LS, it was obvious that ewes carrying the TT genotype and conceiving for the first time in the late favorable season had larger LS. Similarly, Chu et al. (2003) reported that Small Tailed Han ewes with the TT genotype had larger LS. Moreover, Mediterranean Italian buffaloes carrying the TT genotype had a mating period that occurred largely during increasing day lengths and could be allocated to reproduction during long photoperiods, in contrast to the CC genotype (Carcangiu et al., 2011b). The relationship between the variants of the AA-NAT gene and reproductive traits in the first conception season was evaluated in Table 6. The results showed that GG ewes that conceived for the first time in the early favorable season or in the unfavorable season had fewer days to reach age at first lambing. This observation agrees with the previous results of Ding-ping et al. (2012). It is clear from the obtained data that ewes carrying CT or GG genotypes could be allocated for reproduction during long photoperiod months, while ewes with TT or AA genotypes would tend to reproduce during the natural mating season. Overall, our results clarify the complexity of genotype-by-season interactions by showing that the mode of MTNR1A and AA-NAT gene action may differ depending on the season of conception under consideration, probably as a response to fluctuation in melatonin secretion induced by photoperiod. This complex mode of interaction between genetic polymorphisms and the changing season is a vital source of phenotypic variation in reproductive traits. On farms, animals that are less seasonal in their breeding activity should be used in the unfavorable conception season, while seasonal animals should be used in the favorable conception season (Ramírez et al., 2009).

## 5 Conclusion

The presence of genetic polymorphisms in the MTNR1A and AA-NAT loci was confirmed in Egyptian sheep breeds. The results indicated a significant relationship between the MTNR1A and AA-NAT loci and reproductive traits (age at first lambing, lambing interval and litter size). These data demonstrate the importance of both loci, because their polymorphisms could be potential genetic markers suitable for improving the efficiency of reproduction during the unfavorable season in sheep, and the obtained desirable genotypes could be considered in new genetic selection schemes. To the best of our knowledge, this is the first study concerning polymorphisms in the MTNR1A–RsaI and AA-NAT–SmaI loci and their association with reproductive traits in Egyptian sheep breeds. Further studies are required to validate these associations in a larger population.

## Data Availability

No data sets were used in this article.

## Appendix A List of abbreviations

**Table:**

melatonin receptor 1A	(MTNR1A)
Arylalkylamine N-acetyltransferase	(AA-NAT)
PCR restriction fragment length polymorphism	(RFLP)
single nucleotide polymorphism	(SNP)
quantitative trait loci	(QTL)
marker-assisted selection	(MAS)
age at first lambing	(AFL)
lambing interval	(LI)
litter size	(LS)
early favorable	(EF)
late favorable	(LF)
unfavorable	(UF)
